# Assessment of microvascular flow in human atherosclerotic carotid plaques using ultrasound localization microscopy

**DOI:** 10.1016/j.ebiom.2024.105528

**Published:** 2024-12-26

**Authors:** Henri Leroy, Louise Z. Wang, Anatole Jimenez, Nassim Mohamedi, Clément Papadacci, Pierre Julia, Salma El Batti, Jean-Marc Alsac, Jonas Sitruk, Armelle Arnoux, Patrick Bruneval, Emmanuel Messas, Tristan Mirault, Guillaume Goudot, Mathieu Pernot

**Affiliations:** a Physics for Medicine Paris, INSERM U1273, ESPCI Paris, CNRS UMR 8063, PSL Research University, Paris, France; b Université Paris Cité, INSERM U970, Vascular Medicine Department, Hôpital Européen Georges-Pompidou, Assistance Publique-Hôpitaux de Paris (AP-HP), F-75006, Paris, France; c Université Paris Cité, INSERM U970, Vascular Surgery Department, Hôpital Européen Georges-Pompidou, Assistance Publique-Hôpitaux de Paris (AP-HP), F-75006, Paris, France; d Université Paris Cité, AP-HP, Hôpital Européen Georges-Pompidou, Assistance Publique-Hôpitaux de Paris (AP-HP), Clinical Research Unit, Clinical Investigation Centre 1418 Clinical Epidemiology, INSERM, INRIA, HeKA, Paris, France; e Université Paris Cité, INSERM U970, Cardiology Department, Hôpital Européen Georges-Pompidou, Assistance Publique-Hôpitaux de Paris (AP-HP), F-75006, Paris, France

**Keywords:** Atherosclerosis, Ultrasound imaging, Carotid plaque, Neovascularisation, Super-resolution imaging, Contrast-enhanced ultrasound

## Abstract

**Background:**

Neovascularisation of carotid plaques contributes to their vulnerability. Current imaging methods such as contrast-enhanced ultrasound (CEUS) usually lack the required spatial resolution and quantification capability for precise neovessels identification. We aimed at quantifying plaque vascularisation with ultrasound localization microscopy (ULM) and compared the results to histological analysis.

**Methods:**

We conducted a prospective, monocentric, study involving patients who were undergoing carotid endarterectomy (CEA) for carotid artery stenosis. The day before CEA ultrasound examination coupled with the injection of microbubbles (MB) as a contrast agent (CEUS) to image the MB circulating within and around the carotid plaque was performed. CEUS images analysis classified patients into 2 groups: absence of neovascularisation (group A) or presence of neovascularisation (group B). ULM was performed by localising and tracking individual MB centres to reconstruct the neovessels structure with a resolution of around 60 μm. Plaques were manually segmented on the images to quantify the number of neovessels and various haemodynamic metrics inside the plaques. Histological analysis of the excised carotid plaque specimens classified patients into 2 groups: absence of neovascularisation (group I) or presence of neovascularisation (group II).

**Findings:**

Among the 26 patients included, classification was as follows: group I: n = 8 and group II: n = 18, 18 patients had analysable CEUS images and were classified as follows: group A: n = 10, group B: n = 8. The median (Q1-Q3) number of MB tracked per second inside the plaque was 0.03 (0–0.37) for patients in group I and 0.51 (0–3) for patients in group A versus (vs.) 3.55 (1.26–17.68) for patients in group II and 9.69 (5.83–34.68) for patients in group B (p = 0.00049; p = 0.010 respectively). The length of the MB tracks was 0.02 mm (0–0.16) in group I vs. 0.29 mm (0.22–0.45) in group II (p = 0.0069). The study also showed that flow in the neovessels was greater during systole than during diastole period: 9.38 (1.67–19.17) MB tracked per second vs. 1.35 (0.28–6.56) (p = 0.021).

**Interpretation:**

ULM allows the detection of neovessels within the carotid atherosclerotic plaque. Thus, ULM provides a precise picture of plaque neovascularisation in patients and could be used as a non-invasive imaging technique to assess carotid plaque vulnerability.

**Funding:**

The study was sponsored and funded by 10.13039/501100002738Assistance Publique–Hôpitaux de Paris (CRC 1806 APHP INNOVATION 2018). Co-funding by 10.13039/100007383ART (Technological Research Accelerator) biomedical ultrasound program of INSERM, France.

**Research in context**

**Evidence before this study**

Carotid plaque neovascularisation is known to contribute to the vulnerability of carotid plaques and to play a role in plaque rupture as well as other factors such as plaque calcification, and intra-plaque haemorrhage. However, the small size of neovessels has made them very difficult to characterise precisely through conventional (US) or contrast-enhanced ultrasound imaging (CEUS) until now because of the conventional diffraction limit. In this context, ultrasound localization microscopy (ULM), a super-resolution method based on ultrafast ultrasound imaging and the injection of microbubbles (MB) as contrast agents could be a relevant tool to assess the presence of neovessels within the plaque.

**Added value of this study**

We showed that ULM can differentiate plaques with neovessels and plaques without neovessels. After the injection of MB, it can add valuable information to CEUS evaluation of the carotid plaque and additionally, ULM gives access to the blood flow velocities within the plaque. Our study also showed that the MB flow inside the plaque was greater during systole than during diastole.

**Implications of all the available evidence**

ULM could be used to develop new biomarkers associated with the vulnerability of the carotid plaque, paving the way to the development of new methods for non-invasive, non-irradiating and low-cost assessment of the plaque rupture risk.

## Introduction

An important proportion of strokes are attributable to thrombus embolisation from a carotid plaque. Plaques prone to rupture are characterised as vulnerable, combining one or more features, such as intra-plaque haemorrhage, a large necrotic lipid core, inflammation, or neovascularisation.^1^ Such characteristics have been identified by histological analysis of the excised plaque after carotid endarterectomy (CEA). Therefore, detecting and quantifying these vulnerability features in a non-invasive way is of major interest to estimate the risk of ipsilateral stroke in patients and determine the most appropriate therapeutic approach, including intensive medical therapy, possible endarterectomy or stenting.^2^^,^^3^

Until now, precise characterisation of plaque neovascularisation by conventional ultrasound imaging has been challenging due to multiple physical constraints: vessel size below the diffraction limit, small velocities making them harder to distinguish from the surrounding slowly moving tissue, phase aberration and signal attenuation due to plaque calcifications, arterial wall motion, and blood flow signal interference from the arterial lumen. The neovessels are not directly visible by ultrasound with the usual colour or power Doppler modes because of their small size. Contrast-enhanced ultrasound (CEUS) can only provide a semi-quantitative assessment of their presence via the intensification of the grey level of the contrast image after microbubbles (MB) injection.^4^^,^^5^ In previous studies, this plaque enhancement in CEUS mode has been associated with histological microvascularisation.^6^, ^7^, ^8^ It has therefore widely been used to assess the presence of intra-plaque neovessels and, combined with other indicators, to try and refine plaque vulnerability.^9^, ^10^, ^11^ Yet, recent studies pointed out that there was no evidence of a linear correlation between CEUS intensity enhancement and the flow inside the neovascular network inside the plaque, and other mechanisms have been proposed for carotid plaque enhancement, such as direct MB penetration from the lumen inside the plaque or macrophage-mediated transport, suggesting that this enhancement of CEUS signal intensity alone may not be sufficient to give a faithful representation of the neovascularisation of the plaque.^12^, ^13^, ^14^, ^15^ Thus, the evaluation of neovascularisation of the carotid plaque through ultrasound imaging (with or without contrast agents) remains an area of investigation.

In this context, we propose to apply ultrasound localization microscopy (ULM), a contrast ultrasound super-resolution imaging technique, on carotid atherosclerotic plaques to visualise and quantify microvascular flow parameters, such as the amount of MB flowing within the plaque or the velocity of the flow inside the neovessels, which remain mostly unknown. ULM consists of localising at a sub-wavelength scale and tracking the centres of MB used as contrast agents to map the vascular networks of animals and humans in various areas of a wide range of organs and estimate blood velocities in small arteries and veins.^16^^,^^17^ The inherent characteristics of this method, i.e. localisation and tracking, enable it to achieve higher spatial resolution compared to other ultrasound imaging methods and to provide quantitative flow parameters. This has recently been exploited in several clinical applications such as transcranial imaging of the cerebral vascular network in humans^18^ or quantification the activity of arterial diseases such as Takayasu arteritis.^19^ Based on those promising perspectives, this pilot study aims to evaluate the ability of ULM to provide quantitative information about neovascular flow in the context of atherosclerotic carotid plaque instability assessment *in vivo* for patients with severe carotid atherosclerosis.

## Methods

### Population

We conducted a prospective monocentric study at the Hôpital Européen Georges-Pompidou, Assistance Publique-Hôpitaux de Paris, France. Patients were eligible for inclusion if they were aged 18 or older and considered for CEA after assessing their atherosclerotic carotid artery stenosis by duplex ultrasound and angio-computerised tomography or angio-magnetic resonance imaging. Symptomatic patients (with stroke or transient ischaemic attack within 6 months) and a carotid stenosis ≥50% according to the North American Symptomatic Carotid Endarterectomy Trial (NASCET) criteria and asymptomatic patients with a carotid stenosis ≥ 60% were operated on after multidisciplinary team review based on the European Society of Cardiology (ESC) Guidelines.^20^ Patients were excluded if they had non-atherosclerotic carotid stenosis, a contraindication to the use of the ultrasound contrast agent (sulphur hexafluoride microbubbles, Sonovue®, Bracco©), or were unable to give consent to the study.

### Ethics

An independent ethics committee (CPP EST II, 2020.06.02, protocol CNRIPH: 20.03.12.45445) approved the study protocol, under which patients were recruited with *ClinicalTrials.gov* Identifier NCT04470687. All patients provided written consent for the preoperative ultrasound examination and the contrast agent injection. Surgical specimens from the CEA of each patient were analysed in the histology department using standard staining. The study investigators collected clinical, histological, and ultrasound data. The data were collected between October 2020 and June 2022.

### Ultrasound imaging & contrast agent injection

We used an Aixplorer® V9 ultrasound scanner (SuperSonic Imagine©, Aix-en-Provence, France) connected to a 7 MHz central frequency and 200 μm pitch $\left(p_{probe}\right)$ linear probe (SL 10-2) for the examination. A specific ultrafast ultrasound sequence was used to perform imaging: 8 tilted plane waves regularly ranging from −5° to 5° with a PRF of 13 kHz and a frame rate of 500 Hz, a central pulse frequency of 6.42 MHz and a voltage of 8 V. The sequence was calibrated: the mechanical index (MI) was below 0.2 (which is compatible with the use of MB). The in-plane resolution is limited by the pulse wavelength, which, given those parameters, is approximately 240 μm, and the off-plane resolution is limited by the focus in elevation (around 700 μm).

Acquisitions were performed on the common carotid artery and at the carotid bifurcation, at locations where carotid plaques had been identified on patients beforehand with a commercial scanner.

A bolus of sulphur hexafluoride MB (Sonovue®, Bracco©, Milano, Italy) was injected intravenously. The sequence was applied for 8.8 s once the MB were visually detected in the carotid artery lumen using the B-mode image. The radiofrequency (RF) data were stored and processed offline.

### Data processing

**Fig. 1:**
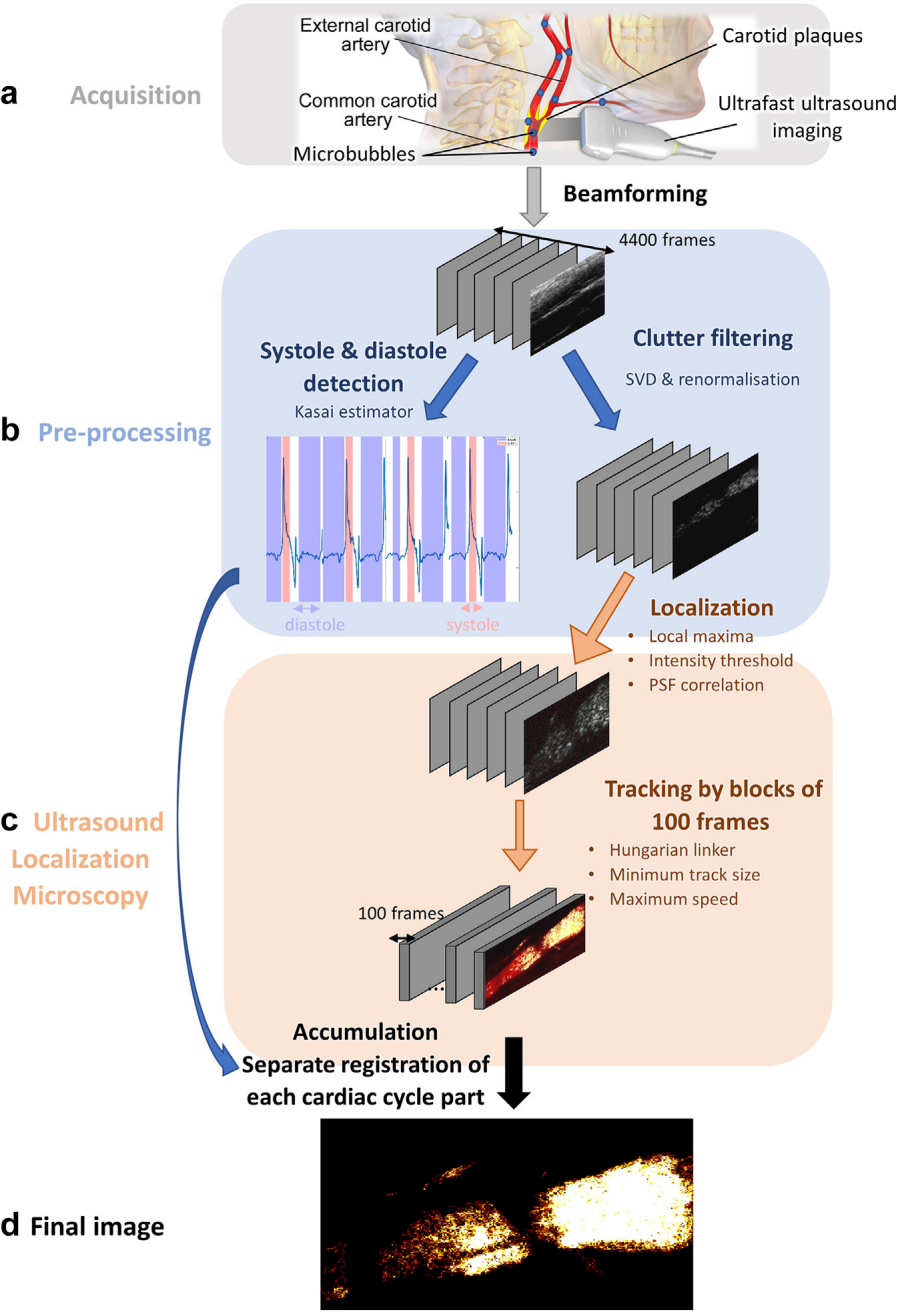
Summary of the processing pipeline for ULM analysis. (a) Acquisition: ultrasound imaging coupled with injection of MB. Radiofrequency (RF) data are beamformed with a delay-and-sum algorithm. (b) Pre-processing of the data. Cardiac cycle parts are detected based on tissue Doppler: the data is divided between systole and diastole periods on which the ULM processing will be applied separately). Clutter filtering based on Singular Value Decomposition (SVD) and signal renormalisation are applied to extract MB signal. (c) ULM processing on each block of filtered frames: localisation of the centres of the MB & tracking of their positions. (d) Rigid registration of the MB tracks' positions for each cycle part and accumulation of the tracks over the whole acquisition.

Fig. 1 summarises the whole processing pipeline. All the post-processing steps were performed using Matlab R2022A® (The MathWorks©, Natick, MA, USA). After in-phase and quadrature (IQ) demodulation, beamforming, and coherent compounding of the different angles, we obtained a series of 4400 reconstructed images. The field of view in the azimuthal direction ($\vec{x})$ was 25.6 mm, and the image was reconstructed in depths $\left(\vec{z}\right)$ between 7 and 35 mm. The pixel dimensions were: $d_{x}=p_{probe}=200\;\mu m$ and $d_{z}=\frac{\lambda }{2}\approx 120\;\mu m$. Logarithmic compression allowed us to visualise the B-mode images. To separate the tissue from the flow, we used spatiotemporal filtering based on singular value decomposition (SVD)^21^ with a fixed ensemble length of 176 frames (i.e. 352 ms) for each spatiotemporal window and a fixed cut-off, filtering out 20% of the singular values to eliminate tissue and retrieve the filtered flow signal (flow Doppler images). The filtered flow Doppler images were normalised by the square root of the temporal mean B-mode signal.

### Motion compensation & registration

The unfiltered B-mode data were used to compute the velocity of the two opposite arterial walls (i.e. tissue Doppler) based on the Kasai autocorrelation estimator.^22^ This relative axial tissue velocity estimation allowed us to visualise the arterial pulsatility and detect systole and diastole periods. The blocks of frames corresponding to those two parts of the cardiac cycle were selected separately using an automated method. This selection allowed motion compensation for breathing, artery motion, and potential probe motion, which was achieved through an intensity-based translation (i.e. rigid) registration applied to each diastole period based on the power Doppler of the normalised filtered flow Doppler signal. The same process was applied to the systole periods.

### Ultrasound localization microscopy and tracking

ULM processing was performed on the blocks of systole and diastole separately using the normalised filtered flow Doppler images divided into numerous blocks of 100 frames, which were processed independently and later rasterised.

First, we localised the centres of the MB. Because MB are much smaller than the wavelength (diameter d ≈3 μm << λ = 240 μm) and distributed in a sparse enough way, MB can be seen as the point spread function (PSF) of the imaging system and their response to ultrasound pressure field makes their signal stronger than their surroundings. Therefore, to detect the MB, we used MATLAB's *imregionalmax* function to spot the local maxima of the image combined with a minimal intensity level of −18dB and a minimal correlation coefficient of 0.60 with an ideal PSF (2D gaussian PSF with standard deviations σ_x_ = σ_z_ = λ/2 = 120 μm). Then, the MB's centres were localised by interpolating the selected intensity maxima profiles.

Second, the localised MB centres were tracked on each block of 100 frames with a frame-by-frame pairing of their positions based on the Hungarian method.^23^ The velocities of the MB that are tracked are computed based on the cartesian coordinates of the centres of the MB and the timestep between two frames. Tracking was further refined considering only the MB with a maximal track velocity of 200 mm/s and a minimum track length of 3 consecutive frames with no gap between frames allowed. These settings allowed the efficient removal of moving tissue signals that might have remained after filtering and noise artefacts that could have been confused with MB.

Eventually, the ULM data were accumulated separately over all the systole blocks and all the diastole blocks, with the positions of pixels corrected based on the registration procedure explained earlier.

A reconstruction of the neovessel's anatomy based on the MB count (density map) and the velocity of the MB (velocity map) is finally obtained on a grid with a pixel size of 60×100μm.

### Plaque segmentation

**Fig. 2:**
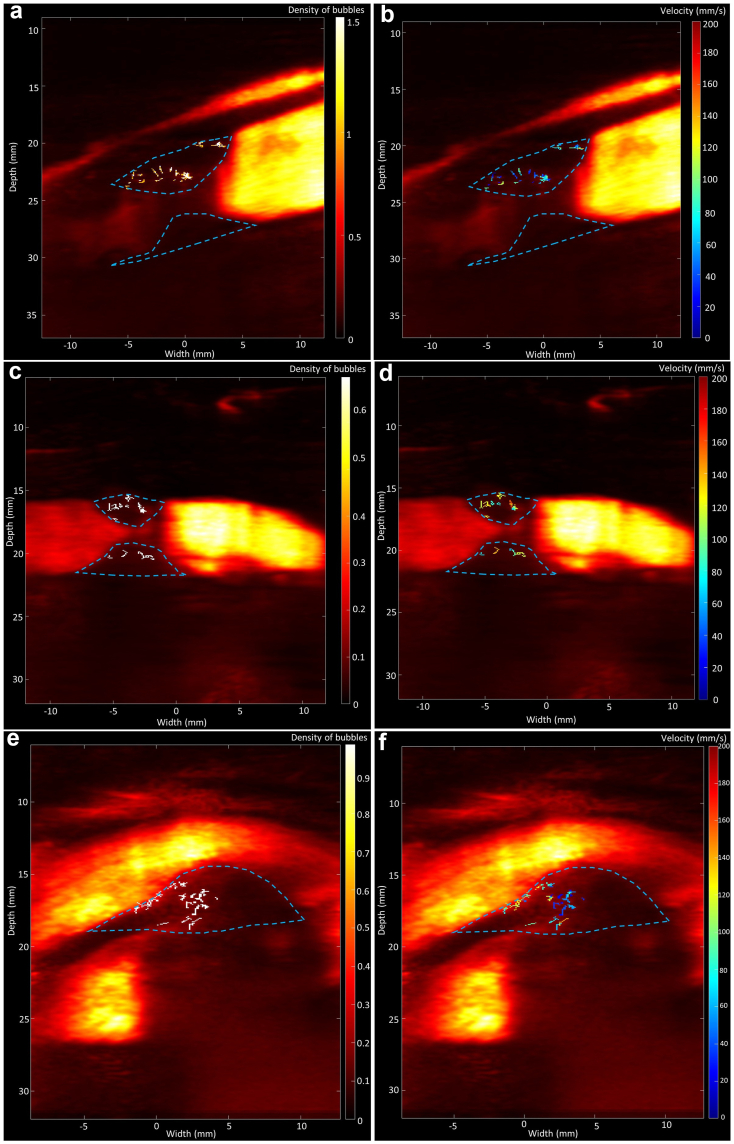
Examples of ULM density and velocity maps of group II (with neovessels) patients with plaque segmentation superimposed.

Based on the Doppler images, a trained medical doctor performed the manual segmentation of the plaques (which might develop either on the lower boundary of the artery or the upper boundary or both). Given the fact that the shape of the artery is quite different during systole and diastole, one segmentation was performed for each cycle part's registered image. Fig. 2 shows examples of ULM density and velocity maps of patients with neovessels detected with this method, with plaque segmentation superimposed onto them.

### Quantitative analysis

Based on our ULM results, we computed various metrics to analyse the results of our processing pipeline. The metrics and scores were computed based on the data of pixels located inside the segmented area representing the carotid plaque, i.e. we associated ULM detections inside the segmentation with neovessels. For each patient, we combined the scores during systole and diastole periods, adding extensive quantities (such as the total number of MB or tracks) and averaging intensive quantities (such as the MB's velocities) with weights based on the duration of each period. We performed a normalisation of the number of MB by the power Doppler inside the segmented plaques to account for the fact that there is not necessarily the same amount of MB inside the carotid of each patient: we computed the scores with and without this normalisation.

We computed the following metrics to evaluate neovascular flow:

- The number of MB tracked per second. Extracted from the density map, it represents the number of MB that are tracked inside the plaque per second of acquisition.
- The average of the MB track lengths inside the plaque. Extracted from the density map, they represent the average length (in number of pixels, then converted in mm) of the tracks identified by the ULM algorithm inside the plaque.
- The MB track velocities. Extracted from the velocity map, they represent the velocities (in mm/s) of the MB that are tracked inside the plaque.

We also computed the mean of the power Doppler signal inside the plaque during the acquisition to see if ULM could provide more relevant or precise information than power Doppler or if the presence of neovascularisation was already visible on Doppler data before any localisation or tracking step.

### Histological analysis

Histological analysis was performed for each patient using cross-sections of the formalin-fixed paraffin-embedded plaque harvested by the surgeon during CEA. Operators trained on the microscopic sections assessed the presence and quantification of neovessels within the plaque. The patients were divided into 2 groups:

- Group I: no neovascularisation visible on histological data
- Group II: neovascularisation visible on histological data

### CEUS acquisitions

For each patient, clinical CEUS images were acquired after the injection of MB and the specific ULM sequence to compare the two methods. The physicians used that set of images to qualitatively classify the patients in one of the two groups (with or without neovascularisation) following the usual CEUS guidelines^24^:

- Group A: no neovascularisation visible on CEUS data
- Group B: neovascularisation visible on CEUS data

This classification did not necessarily follow the histological data classification: this will be discussed later.

### Comparison of perfusion during different cycle parts

As we stressed earlier, the behaviour of the artery is different during systole and diastole; that is why, for each patient, we also decided to compute and compare the scores defined above during systolic and diastolic periods. Given the fact that we accumulate our measure over more than 5 s, we can capture at least 5 complete cardiac cycles (i.e. one systole and one diastole) for each patient and average our measure over this timespan.

### Statistics

The results are presented as follows: median (25th—75th percentile) or percentage (number of patients) to describe quantitative scores or categorical characteristics and outcomes of a given group. We compared the scores between different groups by performing a statistical test to assess the validity of our null hypothesis: “The distribution of scores from the two groups are identical”. Assuming that two groups containing different patients are independent of each other, we used a Mann–Whitney U test to compare the scores between group I and group II or between the group A and group B. For these comparisons, we reported the difference of the medians between the two groups, the difference in location (i.e. the value of the Mann–Whitney test statistic) associated with 95% confidence interval and the p-value. To compare the different cycle periods on the same patients (which are sets of data acquired on the same individuals at different time periods and therefore naturally paired), we used a sign test. For this test, we reported the difference of the medians between the two groups and the p-value. Regarding sensitivity and specificity estimations, we computed Clopper-Pearson confidence intervals. The analyses were performed using MatLab R2022A®software (The MathWorks©, Natick, MA, United States) and R 4.0.3 software (The R Foundation for Statistical Computing).

### Sample size estimation for patient recruitment

For this pilot study, we expected to observe no MB tracks for patients without neovascularisation (group I) and some MB tracks for patients with neovascularisation (group II). Since no previous data on ULM inside the plaque neovessels was available, we decided to hypothesise that the mean number of MB tracks per second of acquisition in patients of group I vs. group II were respectively 1 vs. 10 (i.e. at least an order of magnitude of difference in absolute values between the two groups). Standard deviations were supposed to be the same order of magnitude of the means. Since we expected almost no bubbles in group I with a standard deviation for group I of the order of magnitude of the mean, the common standard deviation is almost 2/3 of the standard deviation of group II. Group I:group II ratio is approximately 1:2 among these populations of patients. To reach a power of 90% to detect a difference in means between the two groups, with a common standard deviation of 7, a Type I error α = 5%, and a Mann–Whitney U test, 36 patients were required (group I:group II = 12:24). To take into account some patients losses, we decided to recruit 40 patients. This number allowed to keep a statistical power high enough for higher dispersions or greater difference in means.

### Role of funders

The study was sponsored and funded by Assistance Publique—Hôpitaux de Paris (CRC 1806 APHP INNOVATION 2018). The sponsor provided help for the study design, authorities approval and performed data monitoring. Co-funding by ART (Technological Research Accelerator) biomedical ultrasound program of INSERM, France.

## Results

### Patients’ characteristics

From October 2020 to June 2022, 40 patients hospitalised in the vascular surgery department for a CEA were included in the study. Four patients were excluded due to incomplete data either in histological analysis (n = 1) or in image acquisitions (resulting from recording errors during acquisitions or corrupted files that could not be analysed) (n = 3). Following post-processing and proofreading of the ultrasound imaging acquisitions by a trained cardiologist, 10 patients were excluded because of poor image quality due to acquisitions not accurately centred on the region of interest or images affected by artefacts such as acoustic shadowing due to the plaque (i.e. a strong attenuation of the ultrasound wave due to its propagation inside the carotid plaque). Acoustic shadowing is a well-known phenomenon that occurs regularly during conventional ultrasound examinations of carotid stenosis and can have an important impact on the quality of the images obtained, as mentioned in the literature.^25^, ^26^, ^27^, ^28^ Ultimately, the samples of 26 patients were available and passed quality control checks regarding both histological and ULM data and were included in the analysis. The clinical characteristics of the 26 patients are shown in Table 1.

**Table 1:** Clinical data of the 26 patients and according to the presence of neovessels in the plaque detected on the histological sections.

Characteristics	All patients (n = 26)	Plaque without neovessels–group I (n = 8)	Plaque with neovessels–group II (n = 18)
Gender (male)—n (%)	19 (73)	6 (75)	13 (72)
Age (years)	75 (±10)	78 (±6)	74 (±11)
Male	74 (±11)	78 (±7)	73 (±12)
Female	77 (±7)	80 (±1)	76 (±8)
BMI (kg/m^2^)	26 (±4)	25 (±3)	26 (±4)
Male	26 (±4)	25 (±3)	27 (±5)
Female	24 (±3)	25 (±3)	24 (±2)
Recent neurological symptoms (stroke and transient ischemic attack)—n (%)	6 (23)	1 (13)	5 (28)
Male	5 (26)	1 (17)	4 (31)
Female	1 (14)	0 (0)	1 (20)
NASCET Degree of stenosis—%			
<60%—n (%)	5 (19)	3 (38)	2 (11)
Male	3 (16)	3 (50)	0 (0)
Female	2 (29)	0 (0)	2 (40)
60–69%—n (%)	1 (4)	0 (0)	1 (6)
Male	1 (5)	0 (0)	1 (8)
Female	0 (0)	0 (0)	0 (0)
≥70%—n (%)	20 (77)	5 (63)	15 (83)
Male	15 (19)	3 (50)	12 (92)
Female	5 (71)	2 (100)	3 (60)
Peak Systolic Velocity—m.s^−1^	3.0 (±1.2)	3.1 (±1.2)	3.0 (±1.1)
Male	3.2 (±1.2)	3.3 (±1.3)	3.2 (±1.1)
Female	2.6 (±0.8)	2.5 (±0.1)	2.6 (±0.9)
Cardiovascular risk factors			
Hypertension—n (%)	21 (81)	7 (88)	14 (78)
Male	17 (89)	6 (100)	10 (85)
Female	4 (57)	1 (50)	4 (60)
Systolic blood pressure—mmHg	140 (±19)	135 (±14)	143 (±19)
Male	143 (±19)	135 (±14)	146 (±20)
Female	134 (±13)	133 (±11)	134 (±14)
Diastolic blood pressure—mmHg	65 (±12)	63 (±5)	67 (±14)
Male	65 (±13)	62 (±5)	67 (±15)
Female	66 (±10)	66 (±2)	67 (±12)
Heart rate–min^−1^	68 (±8)	70 (±10)	68 (±7)
Male	68 (±8)	72 (±11)	67 (±6)
Female	69 (±8)	66 (±5)	71 (±9)
Diabetes mellitus—n (%)	8 (31)	1 (13)	7 (39)
Male	6 (32)	0 (0)	6 (46)
Female	2 (29)	1 (0.5)	1 (20)
Current smoker—n (%)	8 (31)	3 (38)	5 (28)
Male	6 (32)	2 (33)	4 (31)
Female	2 (29)	1 (0.5)	1 (20)
Cardiovascular medications—n (%)			
Lipid Lowering agent	22 (85)	8 (100)	14 (78)
Male	16 (84)	6 (100)	10 (77)
Female	6 (86)	2 (100)	4 (80)
Antiplatelet	23 (88)	7 (88)	16 (89)
Male	16 (84)	5 (83)	11 (85)
Female	7 (100)	2 (100)	5 (100)

Values are expressed as number (%) or mean (±SD).

**Fig. 3:**
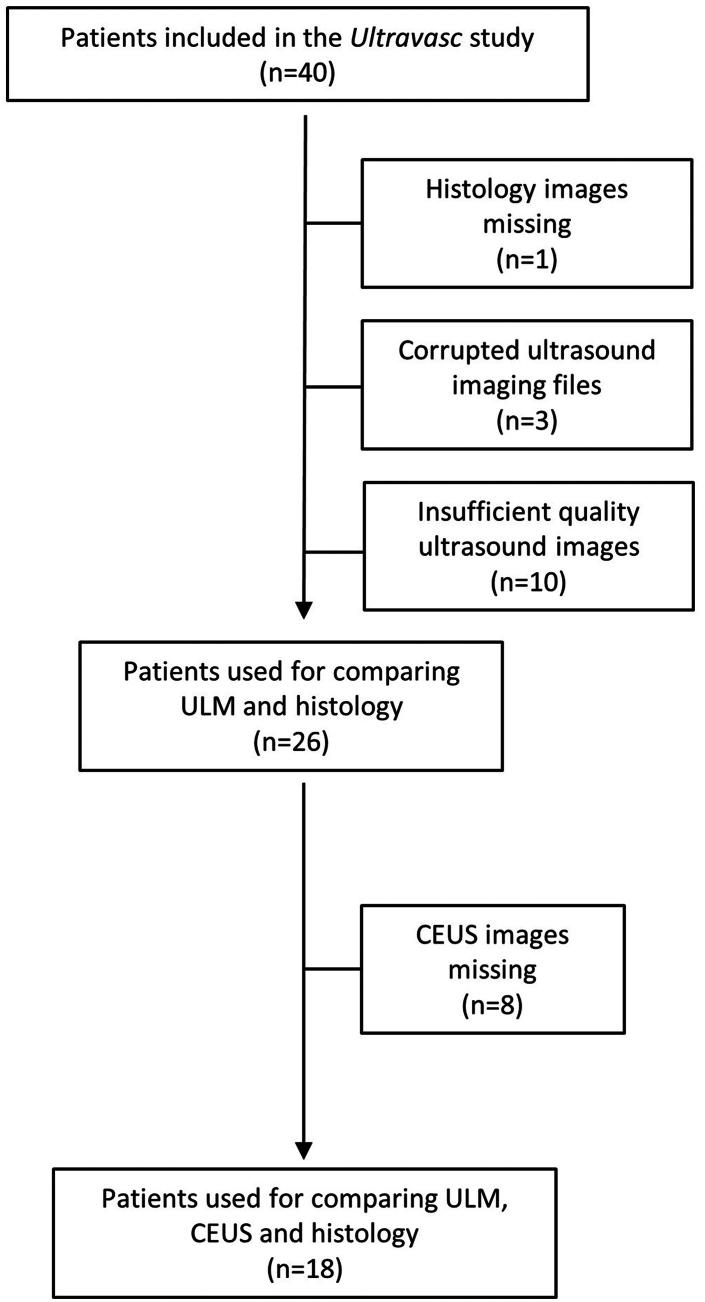
Flowchart describing the inclusion of the patients and the groups used for statistical analysis.

The data from CEUS imaging was not available for 8 patients out of the 26 shown in Table 1. Therefore, the comparative analysis of CEUS and ULM data was performed on 18 patients. The flowchart of Fig. 3 summarises the patients’ inclusion process.

### Multimodal imaging of patients

**Fig. 4:**
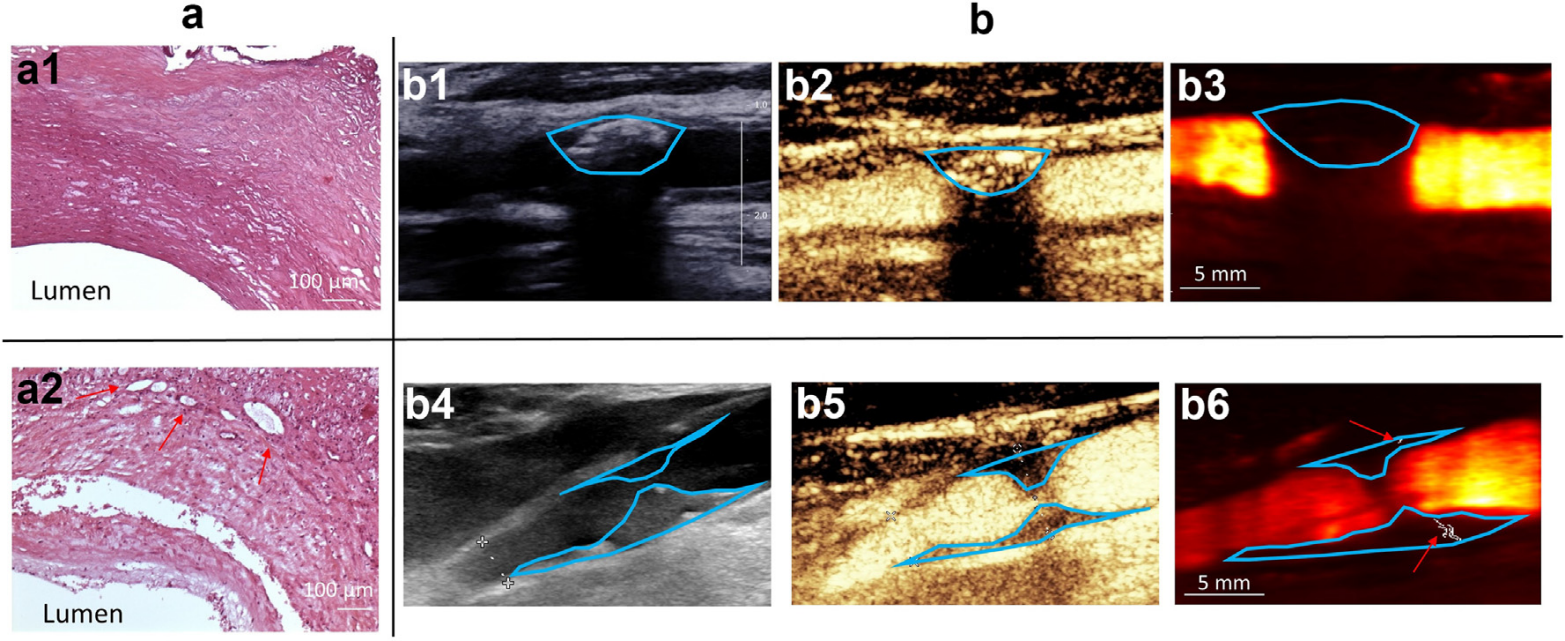
Different imaging modalities in two patients (one from group I (up) & one from group II (down)): (a) microscope transversal view of carotid histological cuts a.1. patient from group I, a.2. patient from group II (red arrows indicate neovessels). & (b) carotid ultrasound images in longitudinal views. b.1. B-Mode image of patient from group I, b.2. Normalised power Doppler of the carotid artery combined with plaque segmentation and ULM map of patient from group I, b.3. Contrast Enhanced Ultrasound image of patient from group I. b.4. B-Mode image of patient from group II, b.5 Contrast Enhanced Ultrasound image of patient from group I, b.6 Normalised power Doppler of the carotid artery combined with plaque segmentation and ULM map of patient from group II (red arrows indicate neovessels).

Fig. 4 shows the B-mode image, the ULM map, the CEUS image and the histological view of a carotid plaque from a patient belonging to both group I & group A (with no MB detected within the plaque with either CEUS or ULM) and the images from a patient belonging to both group II & group B (with a carotid plaque presenting many neovessels, MB being detected in ULM and CEUS).

### Neovessels assessment based on histology

Histological analysis of the 26 plaques revealed that 31% (n = 8) belonged to group I (i.e. no neovessels visible by histology), and 69% of the patients (n = 18) belonged to group II (i.e. neovessels were visible by histology). Most of the plaques with neovessels also presented other vulnerable plaque features (n = 15). The histological characteristics of all plaques and the presence of neovessels are summarised in Table 2.

**Table 2:** Histological data of all plaques and according to the presence of neovessels.

Histological features	All plaques (n = 26)	Plaque without neovessels–group I (n = 8)	Plaque with neovessels–group II (n = 18)
Unstable plaque—n (%)	17 (65)	3 (38)	14 (78)
Male	13 (68)	2 (33)	11 (85)
Female	4 (57)	1 (50)	3 (60)
Large haemorrhage—n (%)	7 (27)	1 (13)	6 (33)
Male	5 (26)	1 (17)	4 (31)
Female	2 (29)	0 (0)	2 (40)
Small or large Thrombus—n (%)	5 (19)	1 (13)	4 (22)
Male	4 (21)	0 (0)	4 (31)
Female	1 (14)	1 (50)	0 (0)
Large lipid core—n (%)	20 (77)	6 (75)	14 (78)
Male	15 (79)	5 (83)	10 (77)
Female	5 (71)	1 (50)	4 (80)
Ulceration of the plaque—n (%)	4 (15)	1 (13)	3 (17)
Male	1 (5)	0 (0)	1 (8)
Female	3 (42)	1 (50)	2 (40)
High degree of inflammation (defined as more than 2 groups of 50 inflammatory cells)—n (%)	6 (23)	0 (0)	6 (33)
Male	3 (16)	0 (0)	3 (23)
Female	3 (43)	0 (0)	3 (60)

Values are expressed as numbers (%).

### Neovessels assessment based on CEUS

According to the physicians, 56% of patients (n = 10) belonged to group A (i.e. no neovessels were visible on CEUS images), and 44% of patients (n = 8) belonged to group B (i.e. neovessels were visible on CEUS images). The clinicians' classification of the CEUS images was compared with the classification based on histological analysis of the removed plaques, as shown in Table 3. If we consider the histology as the reference, this table allows to compute the sensitivity and the specificity of CEUS compared to histology regarding the assessment of neovascularisation (the positive outcome of the examination being the case where a patient has neovascularisation): sensitivity is 62% (95% confidence interval [32%–86%]) and specificity is 100% (95% confidence interval [48%–100%]).

**Table 3:** Comparison of classification by CEUS and histology.

	Presence of neovascularisation in histology (group II)	Absence of neovascularisation in histology (group I)
Presence of neovascularisation in CEUS (group B)	8	0
Male	7	0
Female	1	0
Absence of neovascularisation in CEUS (group A)	5	5
Male	2	4
Female	3	1

### Comparison of ULM results and power Doppler data with histological analysis

**Fig. 5:**
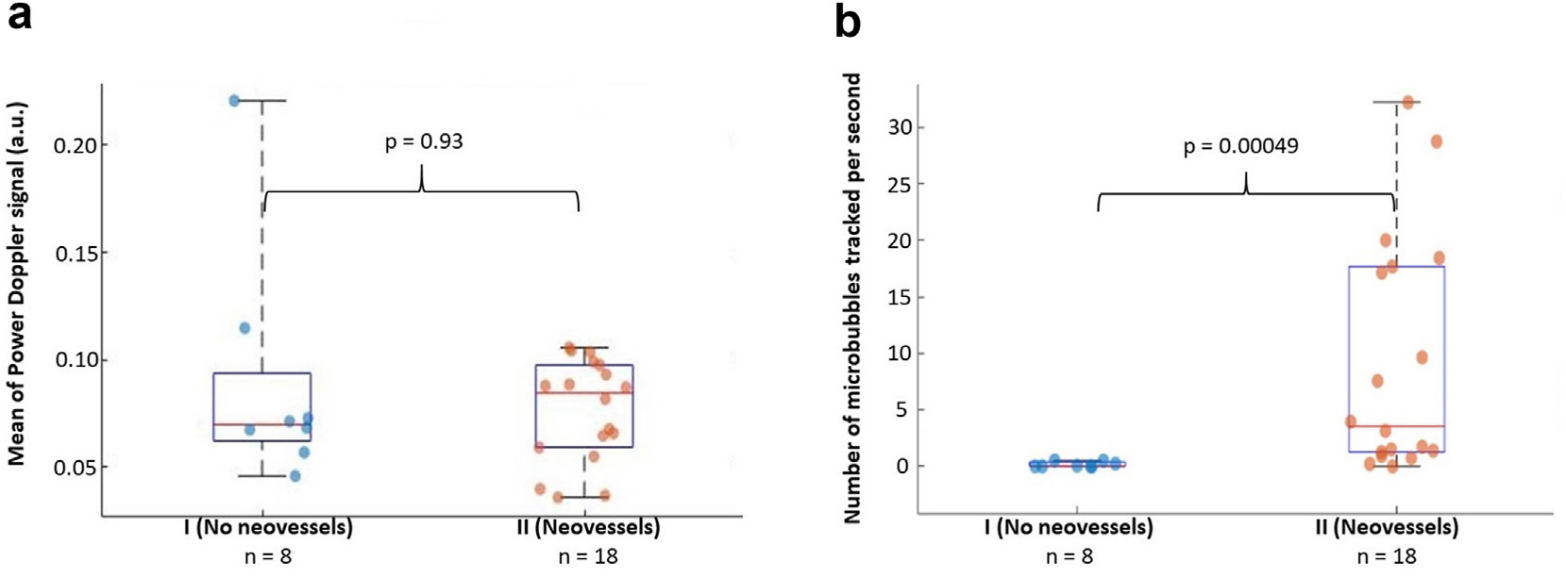
Comparison of the performance of ULM and Power Doppler for the classification of patients based on histological assessment of the neovascularisation (n = 26—Mann–Whitney U test): (a) Mean of Power Doppler inside the plaques (p = 0.93), (b) Number of MB tracked per second inside the plaque (p = 0.00049).

Fig. 5 shows the results obtained with power Doppler data on the cohort of patients compared with ULM results.

On the one hand, it is impossible to conclude that the two groups are different based solely on the mean of the power Doppler signal inside the segmented plaques: the mean power Doppler intensity in group I is 0.070 (0.062–0.094) vs. 0.084 (0.059–0.097) in group II (p-value of Mann–Whitney U test for comparison = 0.93). The differences of the medians between group II and group I is 0.014 and the difference in location is −0.0018 (95% confidence interval [−0.031; 0.026]).

On the other hand, ULM provides information that reasonably allows to separate the two groups:

- the number of MB tracks per second of acquisition was different between the two groups: 0.027 (0–0.37) for patients in group I and 3.55 (1.26–17.68) for patients in group II (p-value of Mann–Whitney U test for comparison = 0.00049). The difference of the medians between group II and group I is 3.52 and the difference in location is 3.37 (95% confidence interval of [0.98; 17.62]).
- the average length of the MB tracks was also different: 0.021 mm (0–0.16) in group I vs. 0.29 mm (0.22–0.45) in group II (p-value of Mann–Whitney U test for comparison = 0.0069). The difference of the medians between group II and group I is 0.27 and the difference in location is 0.25 (95% confidence interval of [0.09; 0.36]).

### Comparison of ULM results with CEUS classification

**Fig. 6:**
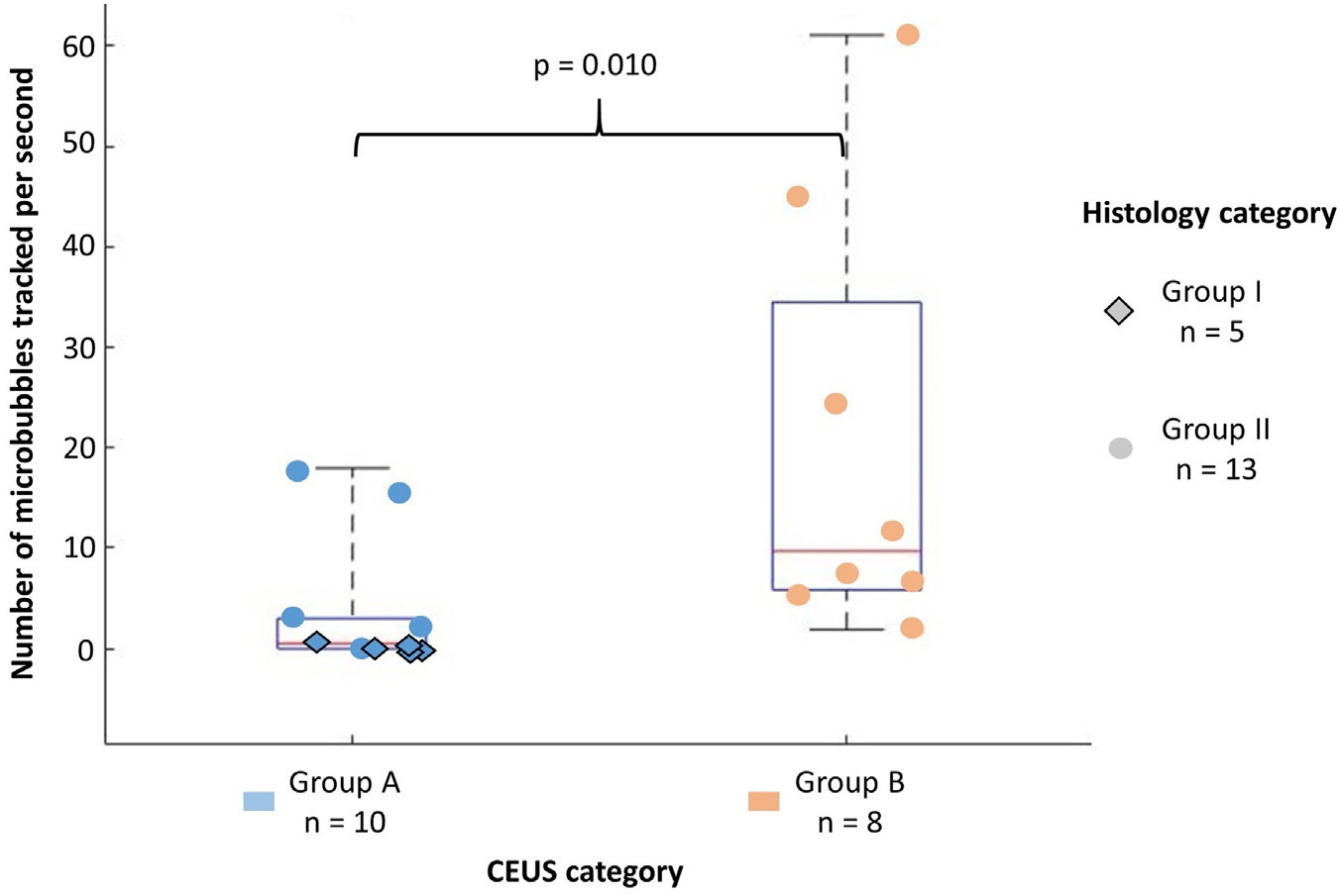
Comparison of three ways of evaluating the presence of neovessels inside the plaque (n = 18—Mann–Whitney U test—p = 0.010): the classification of patients based on CEUS is on the horizontal axis and is indicated by the marker colour, the ULM scores are on the vertical axis, and the classification of patients based on histology is indicated by the marker shape.

As shown in Fig. 6, the number of MB tracks per second of acquisition was different between the two CEUS groups: 0.51 (0–3) for patients in group A and 9.7 (5.8–34.7) for patients in group B (p-value of Mann–Whitney U test for comparison = 0.010). The difference of the medians between group B and group A is 9.19 and the difference in location is 8.91 (95% confidence interval of [0.42; 28.12]).

### Estimation of flow velocities

**Fig. 7:**
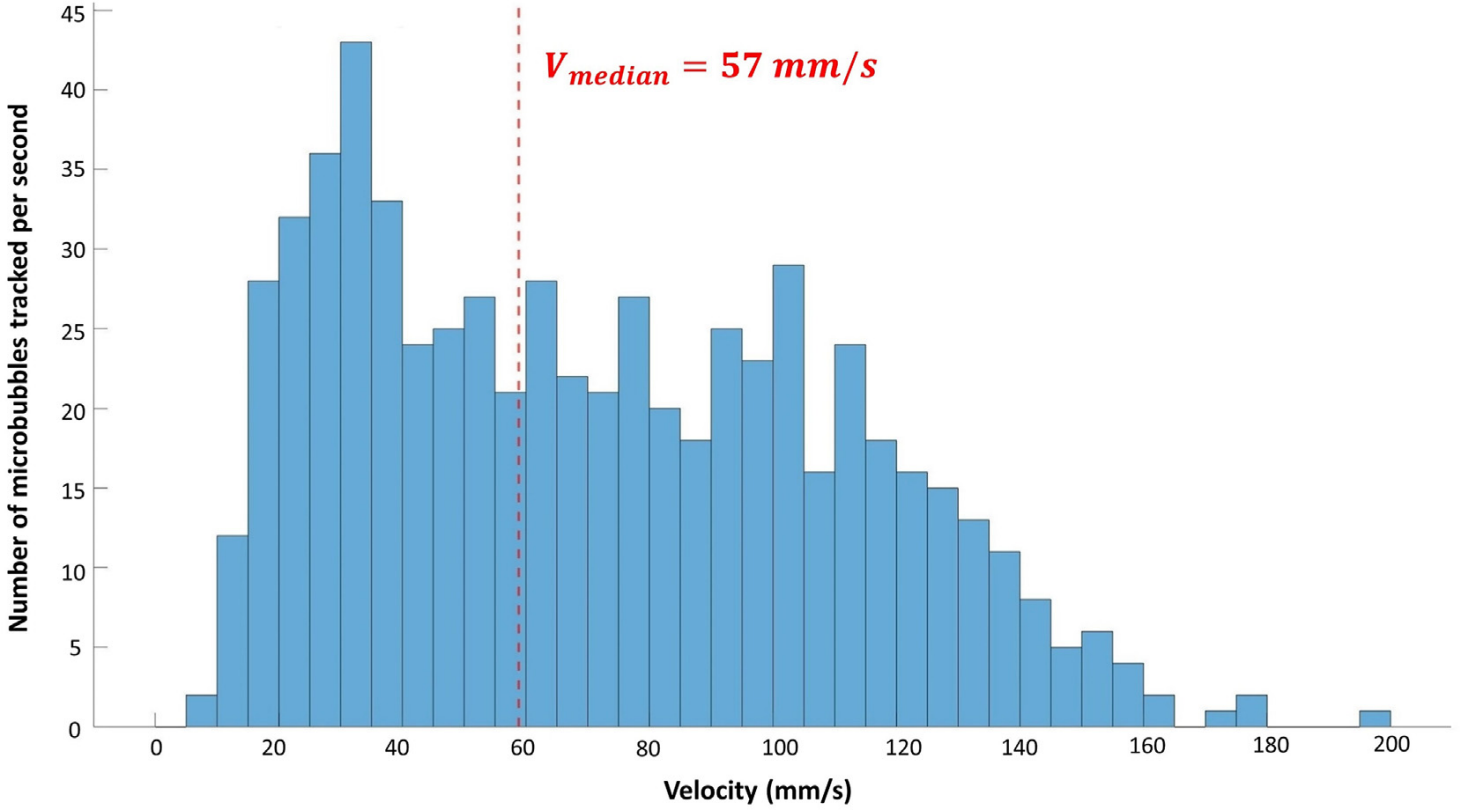
Distribution of velocities of the MB obtained by ULM inside the plaques of group II patients (n = 18).

ULM allows retrieving the axial ($v_{z}$) and lateral ($v_{x}$) components of the MB velocities and their norm. In the plaques of patients from group II, the MB velocities magnitudes mostly ranged from 10 to 160 mm/s with a median (Q1-Q3) velocity of 57 mm/s (33–88) as shown in Fig. 7.

Since patients from group I are not supposed to have neovessels and that, indeed, very few bubbles were detected in those cases, the velocities are mostly measurement noise and don't offer relevant physiological information.

### Differentiated passage of MB between systole and diastole

**Fig. 8:**
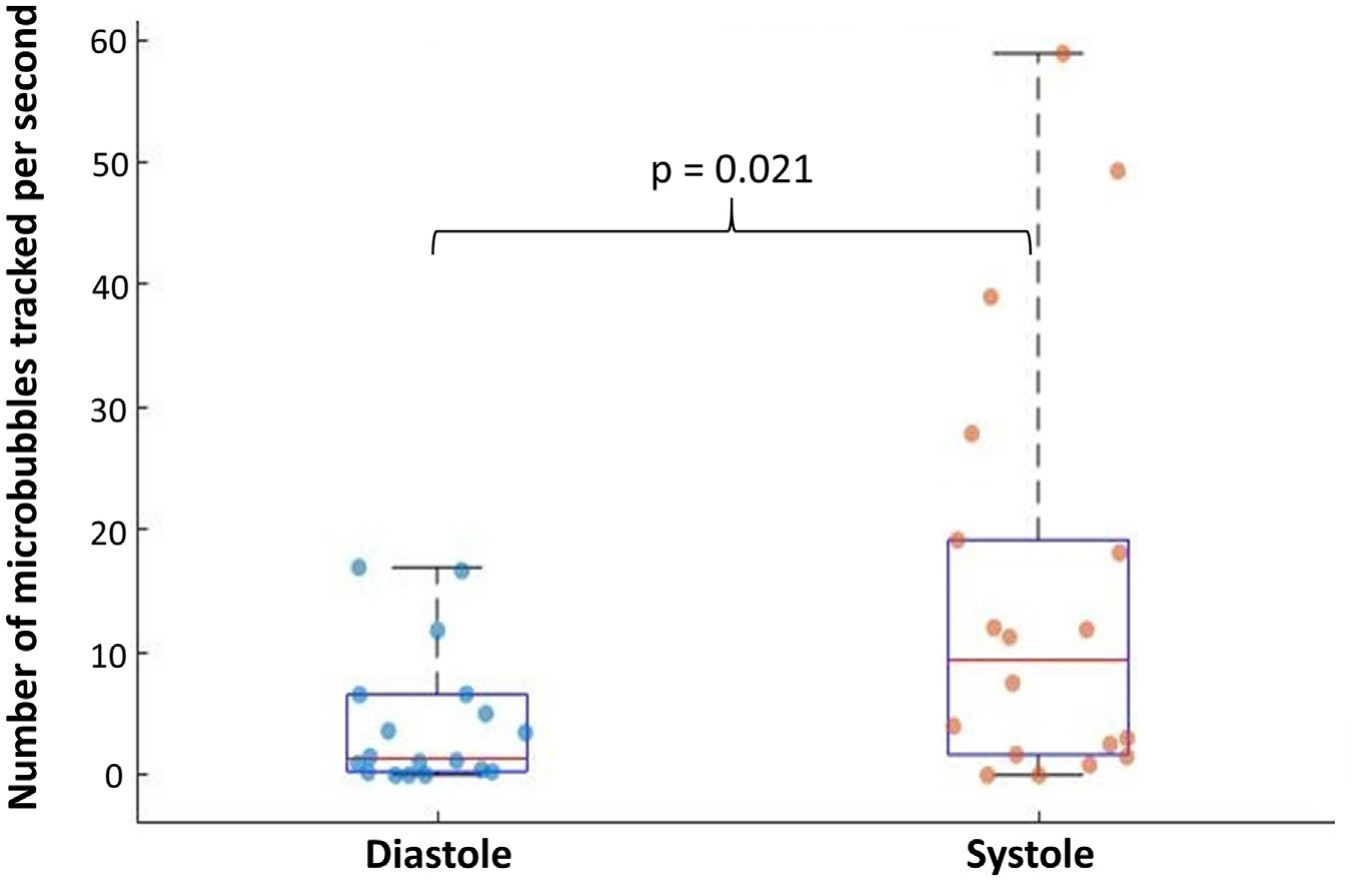
Comparison of number of MB tracked per second inside the plaque during systole and diastole for group II patients (n = 18—Sign test—p = 0.021).

In the plaques of patients from group II, the number of MB tracks per second of acquisition was different between the two cycle parts: 9.38 (1.67–19.17) during systole vs. 1.35 (0.28–6.56) during diastole. The difference of the medians between group II and group I is 8.03 and the p-value is 0.021 (sign test), as evidenced in Fig. 8. The length of the tracks was quite similar: 0.35 (0.21–0.49) mm during systole vs. 0.32 (0.23–0.45) mm during diastole. The difference of the medians is 0.03 and the p-value is 0.25 (sign test). Since patients from group I are not supposed to have neovessels and that indeed, very few bubbles were detected in those cases, the comparison of the number of MB tracked between systole and diastole doesn't offer relevant physiological information.

## Discussion

In this study, we imaged the microvascular flow in carotid atherosclerotic plaque using ULM. Ultrafast ultrasound imaging data were acquired on patients after MB injection and before surgical plaque removal. As opposed to CEUS and the analysis of intensity enhancement performed in previous studies,^5^, ^6^, ^7^, ^8^ our approach was based on localising and tracking the flow of MB injected in the vasculature. These steps, which were performed automatically, allowed the determination of individual MB tracks and quantified parameters associated with the microvascular flow, such as the number of MB tracked per second, the length of the tracks, or the velocity of the MB, to enable the direct characterisation of the neovascularisation. Metrics associated with these parameters were confronted with the classification of the plaques based on histological analysis of the removed plaques, which is currently the *gold standard* method for the evaluation of neovessels but has the disadvantage of being invasive.

The ULM results lift the ambiguity associated with the sole power Doppler signal, which could not discriminate between the different categories accurately. As they are associated with the distribution of velocities, the ULM scores indicate a greater MB flow inside the plaque for patients with neovessels visible on histological cuts.

We also compared these results with CEUS imaging, which is the actual non-invasive reference method.^8^, ^9^, ^10^, ^11^ We observed that the cases where CEUS and histology classifications are not in accordance are exclusively cases where CEUS does not detect neovessels while histology does: the sensitivity of the method is therefore not optimal. Moreover, the wide 95% confidence intervals of both sensitivity and specificity indicate the imprecision of these estimates, which underlines the fact that the reliability of the evaluation of neovessels solely through CEUS is open to question. Our analysis is consistent with previous literature regarding the limitations of the assessment of plaque neovessels with CEUS.^12^, ^13^, ^14^, ^15^ In those cases, since both methods require ultrasound examination coupled with the injection of MB, the ULM score can provide additional information to CEUS to evaluate more precisely the presence of neovessels inside the plaque.

Interestingly, ULM analysis unveiled a different behaviour of the neovessels in the two parts of the cardiac cycle. The study shows that the lengths of the tracks are similar in systole and diastole, which seems logical given that we are comparing the flow of MB inside the neovessels of the same patients at different time steps. These results also indicate a greater number of MB tracked per second within the plaques during systole than diastole period. Since those two parts of the cardiac cycle closely follow each other, one can assume that the concentration in MB of the blood is approximately constant between one systole and the following diastole (and vice versa): this suggests an increase of the blood volume inside the neovascular network of the plaque during systole. This phenomenon might be of interest to physicians to better characterise the development of intra-plaque neovascularisation.

This study has several limitations. The first visible limitation of the method is the low number of MB tracked. For a sequence lasting less than 10 s like ours, this does not allow the rasterisation step to reconstruct the vessel anatomy efficiently. It might later hinder the method's ability to quantify various metrics based on a statistical analysis of the MB's parameters. Implementing longer acquisitions lasting several minutes might be a way to address this issue and accumulate more MB. Those longer acquisitions could also be beneficial to the analysis of the difference of behaviour of the neovessels between systole and diastole periods, since it would allow to estimate the amplitude of this effect with more sample points, increasing its accuracy and making it less sensitive to artefacts or regression to the mean effects.

Other limitations can be found in the design of the clinical study: the number of patients from whom data were quantified is small, which usually means that the power of the statistical analysis is decreased. However, the small number of patients did not prevent us from demonstrating the proof of concept of neovessels imaging with ULM and quantification with notable differences between the different categories of plaques. A study of a wider number of patients as well as a more in-depth analysis of potentially undetected interactions between variables (e.g. physiological parameters and ULM scores) between the different groups could be carried out to assess more precisely the ability of this method to discriminate between various types of patients. Moreover, the population selected for the study was very specific, since the patients who underwent this examination were addressed for CEA, meaning their plaque was probably more prone to develop neovessels than the average plaque. In addition, as stated earlier, some patients had to be excluded from the analysis because of acoustic shadowing. This effect was awaited according to the literature since it is a common challenge for ultrasound examinations of the carotid plaque.^25^, ^26^, ^27^, ^28^ It might be a challenge to try and tackle in future studies. Yet, if this method is generalised on patients with less severe atherosclerosis, the plaques should be thinner or less heterogeneous, leading to less shadowing and, therefore, better quality data.

**Supplementary Figure S1:**
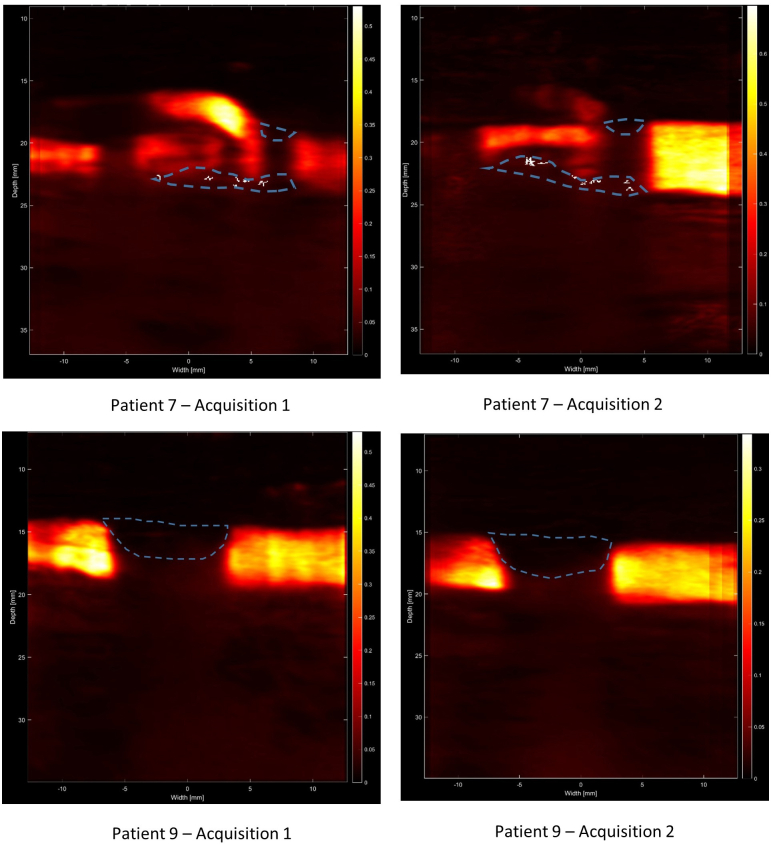
Examples of 2 ULM maps coming from 2 different acquisitions performed on the same patients. The segmentation of the plaque was performed by a trained MD on the B-mode images. Although the position of the probe has clearly moved between the two acquisitions, we can notice that both the power Doppler and the ULM detections show relatively similar patterns—in both a case where neovessels are detected and a case where neovessels are not detected (confirmed by histology).

Additionally, the analysis is based on 2D images, which are highly operator-dependent because they rely heavily on the choice of the acquisition plane. We acquired a couple of acquisitions twice on the same patients in order to try and evaluate the reproducibility of the method (see Supplementary Figure S1): although the position of the probe has clearly moved between the two acquisitions, we can notice that both the power Doppler and the ULM detections show relatively similar patterns. Also, these 2D images might lead to underestimating the velocity of the flow due to the absence of the off-plane component of the velocity and an incomplete estimation of the neovessels’ geometry due to the thinness of the elevation direction. This might prevent us from retrieving a whole vessel as it goes out of the reach of our 2D ultrasound probe.

Eventually, arterial motion becomes a serious obstacle in the precise detection of the neovessels, as it disturbs the filtering operations. We chose to use spatiotemporal filtering based on SVD, but other techniques, such as sliding SVD with a variable ensemble length,^29^ could be used to improve the separation of tissue and flow. Arterial motion might also create artefacts during the rasterisation step (e.g. duplication of neovessels, neovessels cut in the middle). We used a rigid registration based on the intensity of the power Doppler image. However, various techniques such as non-rigid registration or other methods used to perform registration in time over the whole cardiac cycle or in space might improve the rasterisation step in this context.

First, a 3D analysis would have the advantage of a less operator-dependent evaluation of probe positioning. 3D ULM could be applied to solve this issue and provide 3D velocity estimates and flow rates and perfusion inside the plaque. A volumetric acquisition would also give access to the MB perfusion rate within the whole arterial wall, a parameter not accessible on a 2D analysis.

Second, an estimation of the flowrate inside neovessels through ULM in atherosclerotic patients, combined with other parameters such as elastography measurements and wall shear stress estimations to create a clinical score could prove to be a valuable tool for non-invasive evaluation of the vulnerability of the plaque.^4^

Third, clinical studies on different types of patients (e.g. patients not addressed for CEA) could take place to try and validate the method in other contexts and on larger numbers of patients.

Fourth, a method to compensate for aberration and attenuation through the calcified plaques could be implemented to make the data less noisy and, therefore, more reliable.

Eventually, if this method demonstrates its relevance and robustness in different clinical contexts, a translation to clinical practice could be considered through the development of software integrated into the ultrasound scanner, with an automated live imaging mode for the beamforming, compounding, filtering, and localisation steps at least.

In conclusion, ULM is a promising method for evaluating neovascularisation within the atherosclerotic carotid plaque. It allows quantification of MB passage and, thus, the presence of plaque neovascularisation, which is associated with instability of the arterial plaque. This could be an essential biomarker for monitoring therapy designed to reduce plaque inflammation and vulnerability.

## Contributors

T.M., G.G., C.P., and M.P. conceived and supervised the study. G.G., L.W., J.S. and N.M. collected the data. P.J., S.E.B. and J-M.A. were responsible for the carotid endarterectomy of the patients. P.B. performed the analysis of the surgically removed plaques. H.L., A.J. and M.P. performed post-processing of the imaging data. H.L. performed the statistical analysis. H.L., G.G., L.W. and M.P. wrote the manuscript. All authors interpreted the data, drafted, and revised the manuscript, and approved the final version. H.L., G.G., T.M. and M.P. were responsible for the decision to submit the manuscript.

## Data sharing statement

Data that support the findings of this study will be available from the corresponding author of the study.

## Declaration of interests

TM has received honoraria from Novartis, Amarin, Novo-Nordisk and support for attending meetings and travel from Amgen, Bristol Myers Squibb-Pfizer and MSD. TM participates on the board of APHP ITAC trial. The other authors declare no conflicts of interest.
